# From dependency to child maltreatment? Changing frames in U.S. newspaper coverage of welfare policy

**DOI:** 10.3389/fsoc.2026.1860911

**Published:** 2026-09-21

**Authors:** Logan Gray, Stephanie Moller

**Affiliations:** 1 Clinical Rehabilitation and Mental Health Counseling, The University of North Carolina at Chapel Hill, Chapel Hill, NC, United States; 2 Department of Sociology, University of North Carolina at Charlotte, Charlotte, NC, United States

**Keywords:** child welfare, culture of poverty, frame analysis, media, social welfare

## Abstract

**Inroduction:**

Prior research has extensively documented dependency framing in welfare discourse during the mid-to-late twentieth century, but less is known about whether this framing persists in more recent periods. This study examines U.S. newspaper discourse on social welfare during three periods marking key welfare and child welfare policy shifts: 1994–1996 (welfare reform), 2016–2018 (child welfare reform), and 2021–2023 (post-pandemic).

**Methods:**

We randomly sampled 40 ProQuest-indexed newspaper articles from each period (120 total) addressing welfare, child welfare, unemployment insurance, or education spending. Using a mixed deductive-inductive qualitative content analysis, we examined recurring frames, their relationships, and how they were articulated across policy domains and periods. Our analysis characterizes how these frames organize meaning and share an underlying cultural logic rather than estimating their prevalence in the broader population of news coverage.

**Results:**

Consistent with prior research, dependency framing was common in the 1990s sample. Dependency became less prominent relative to child maltreatment framing in the later periods. The policy program under discussion also shaped which frames were most salient. Despite the declining prominence of dependency language, classic culture of poverty logics (individual pathology and family blame) persisted across periods.

**Discussion:**

Moralized narratives associated with culture of poverty theory were rearticulated in forms that increasingly centered children’s endangerment. These findings demonstrate how longstanding cultural logics can persist even as their language and policy focus change, with implications for the construction of deservingness in social welfare debates.

## Introduction

The news media shapes public discourse by deciding which issues become salient and how they are framed (Gamson et al., 1992; de Goe, 1996; Rose and Baumgartner, 2013; Epp and Jennings, 2021). Framing theory suggests that framing selects elements of reality, defines problems, assigns moral judgment, and suggests remedies (Entman, 1993). Media framing is also tied to agenda-setting in the political arena (Nelson, 1986). This study examines how newspaper coverage of social welfare in the United States has shifted across three periods—1994-1996, 2016–2018, and 2021-2023—and which co-occurring frames accompanied the shift. These periods capture the last major wave of welfare reform, child welfare reform, and the sociopolitical transformations surrounding the COVID-19 pandemic. We analyze a sample of 120 articles (stratified by period) and conduct a qualitative frame analysis focused on recurring themes and their thematic pairings that were identified through both inductive and deductive coding. Our focus is on the salience of frames, not on interpreting the micro level semantics or power dynamics of language. In other words, we qualitatively analyze the articles to trace recurring frames, rather than performing a discourse analysis of what the language means or how it works.

Prior research finds that dependency, or reliance on the government for cash assistance rather than individual or family wages (Lewis, 1959; Moynihan, 1965; Lewis, 1966b; Fraser and Gordon, 1994), is historically a central component of media framing of social welfare in the United States (Misra et al., 2003). The notion of dependency emerged in Culture of Poverty theorizing that links poverty to behaviors like out of wedlock births, joblessness, and substance use, behaviors portrayed as culturally entrenched individual failings. Yet less is known about whether this dependency-centered framing persists as welfare and child welfare policy have evolved in more recent decades. This study addresses three research questions: What cultural frames organize newspaper coverage of social welfare programs across the three sampled periods? Which frames co-occur with the dependency frame, and how does that co-occurrence pattern change across periods? How, if at all, does the salience of culture-of-poverty-related framing shift across periods and policy domains?

We find, in alignment with previous research, that dependency is a widespread frame during the 1994–1996 period, but recent newspaper articles widely recast discussions of supportive state policies through a child maltreatment lens, depicting poor parents as neglectful and justifying heightened state intervention. We argue that although early culture of poverty debates did not foreground maltreatment, the shifting focus on maltreatment centers on familial dysfunction and behavioral failing, in alignment with historical cultural theories of poverty. Because dependency and child maltreatment are the two most common culturally related frames in our sample, other frames associated with these frames are analyzed as co-occurring frames. This allows the researchers to identify a shift in the media’s cultural problem definition, from economic dependence to parenting behavior, both variants of culture of poverty reasoning. These frames are discussed in light of the broader discourse.

Newspaper articles continue to exert considerable influence on public discourse despite declining print circulation. In 2022, nearly 21 million daily newspapers remained in circulation, and newspaper websites attracted approximately 10 million unique visitors each month (Naseer and St. Aubin, 2023). Moreover, individuals who primarily access news through social media are often influenced by newspaper articles, as these stories are frequently shared across digital platforms. For instance, a 2010 New York Times blog reported that a Times article link was tweeted once every 4 s (Harris, 2010). Further, many social media influencers who shape public narratives today are directly affiliated with traditional print media outlets (Stocking et al., 2024). Despite evolving patterns of news consumption, content originating from established newspapers remains influential in shaping public discourse. Thus, this research examines the content of newspaper articles.

## Cultural theories of poverty

Cultural explanations of poverty and other forms of destitution have a long history in academic discourse. Oscar Lewis coined the term “culture of poverty” to describe Mexican and Puerto Rican families living in poverty (Lewis, 1959, 1966a, 1966b). He argued that although poor families loved their children and desired to provide opportunities through education, their efforts were often futile due to their subcultural conditions, characterized by promiscuity, single motherhood, unemployment, illness, alcoholism, child abuse, dependency, and a sense of inferiority. They chose immediacy over self-improvement and taught their children to do the same. Lewis acknowledged that these subcultural traits developed in adaptation to social, economic, and political institutions that failed the poor, blamed them for their destitution, and created conditions that isolated them from mainstream cultural values and economic opportunities. He also emphasized that these conditions were reinforced through socialization, perpetuating a cycle of poverty.

Lewis’ Culture of Poverty perspective was implicitly embraced by Daniel Patrick Moynihan, an influential government bureaucrat who served under the Kennedy, Johnson, and Nixon administrations, and ultimately served as a U.S. Senator from 1977 to 2001. Moynihan is perhaps best known for The Moynihan Report (1965) in which he argued that the breakdown of the African American family was a key driver of extended, intergenerational poverty and dependence on public assistance. Moynihan attributed this breakdown to the insufficient assimilation of black males into the labor force, an increase in single mother families, and a rise in illegitimacy. Moynihan acknowledged that poverty was rooted in slavery, segregation, and other social and economic conditions; however, he emphasized that the extended, intergenerational nature of poverty and dependence on governmental assistance resulted from the pathologies among poor families (Fraser and Gordon, 1994; Wilson, 2009b).

Moynihan was criticized by scholars who prioritized structural over individual explanations of poverty. Scholars argued that both Moynihan and Lewis conflated symptoms with causes, engaged in victim blaming, and rationalized oppression (Ryan, 2010; Gans, 2011; McDermott and Vossoughi, 2020). As a result, a sizable body of research emerged to assess the claims of pathology (Gans, 1995; Small et al., 2010; Katz, 2013; McDermott and Vossoughi, 2020). Because of the widespread evidence, the culture of poverty thesis was widely rejected and considered antiquated among academics by the 1980s.

Yet, scholars reinvigorated cultural discussions of poverty during the 2000s. The new scholarship concedes that the original culture of poverty thesis blamed the victim and failed to account for the central role of structural factors, including material deprivation. Revamped cultural theories instead emphasize that even among the poorest communities culture is heterogeneous; people facing the same structural constraints make different, culturally informed choices; and these adaptive practices are often misread and stigmatized by mainstream observers and even by the very networks and institutions on which poor residents depend (Lareau, 2003; Harding, 2007; Smith, 2007; Wilson, 2009a; Small et al., 2010; McDermott and Vossoughi, 2020; Keddell, 2023). In this view, culture operates as a pragmatic adaptation and a resource for resilience rather than a source of pathology.

### Dependency in discourse

Early culture of poverty writers treated dependency as a central problem. Dependency is a highly stigmatized and moralized concept. The term suggests that able-bodied individuals have become dependent on the government for social support due to their own behavioral and moral failings, leading to intergenerational transmission of poverty and welfare receipt (Fraser and Gordon, 1994; Misra et al., 2003). As a result, they are framed as undeserving of support in public discourse. Yet, the boundaries of the dependency frame shifted over time. In the early 20th century, the dependency frame was employed to discuss unemployed men seeking assistance through programs such as unemployment insurance; by the middle 20^th^ century, the dependency frame was primarily invoked to discuss programs that served single parent families, notably social welfare (Misra et al., 2003). This makes sense: in the 1920s and 1930s, women were valued chiefly for domestic labor and deemed deserving of support to care for their families, while able-bodied men who sought aid were stigmatized. By the 1960s, prevailing norms recast women as potential wage earners, and mothers were normatively redefined as able-bodied workers and, like men before them, were reclassified as undeserving (Mink, 1995). This normative shift was most relevant to white women, as Black women were historically excluded from the image of the deserving mom who deserved access to welfare. As more Black women gained access to welfare, welfare dependency discourse emerged (Mink, 1995; Hancock, 2004).

Numerous studies have examined the discourse of dependency in the media, finding a clear and sustained framing of welfare recipients as dependents (Fraser and Gordon, 1994; Bullock et al., 2001; Schram and Soss, 2001; Lens, 2002; Misra et al., 2003; Rose and Baumgartner, 2013; van Doorn, 2015; Epp and Jennings, 2021). Studies have also found that media portrayals of dependents and stigmatized welfare recipients are consistently racialized (Clawson and Trice, 2000; van Doorn, 2015; Abramovitz, 2017). Over the past decade, a growing body of content-analytic scholarship has turned specifically to media narratives about child welfare and maltreatment (Hove et al., 2013; Han et al., 2019; Madden et al., 2021; Yelderman et al., 2022). Scholars have found that thematic framing often depicts a failing system rather than the social and structural conditions underlying maltreatment (Han et al., 2019). The present study extends the literature by illustrating how dependency framing, a foundational frame in culture of poverty arguments, has become less prominent relative to child maltreatment framing, in part because media discussions have shifted from welfare to child welfare.

### From dependency to child maltreatment framing

Child maltreatment was not centralized in early culture of poverty writings. It was only one among many symptoms of a maladaptive subculture in Oscar Lewis’ culture of poverty thesis, and Daniel Patrick Moynihan ignored child maltreatment entirely, portraying poor children primarily as casualties of family breakup rather than parental violence. Even so, Moynihan’s framework helped normalize a broader public behavioral scrutiny of poor families, scrutiny that evolved over time from concerns about economic dependence to concerns about parental neglect and child welfare. This shift did not abandon the culture of poverty thesis. It applied the same moral lens to a different symptom of parental deficiency.

Although the public focus shifted from dependency and public welfare in general to child maltreatment and child welfare, the role that child welfare played in policing poor families was not new. Long before the modern child welfare system took shape, poor families were subject to intensive monitoring by charities, churches, and state actors, who routinely evaluated parents’ morality, work habits, and home life (Pelton, 1978; Katz, 1986; Nelson, 1986). For more than a century, poor children were removed from their homes and placed in orphanages and foster homes. Family breakup was justified as child rescue and rehabilitation (Pelton, 1978; Katz, 1986; Nelson, 1986). Scholars have convincingly argued that child welfare governance did not begin as a response to violence; it began as a response to poverty and to elite judgments about the proper organization of family life (Nelson, 1986).

During the mid–late twentieth century, child maltreatment rose on the public agenda in ways that intentionally divorced child harm and poverty. Early public discussions were anchored in a highly publicized medical study on the “battered child syndrome” which framed child abuse as a clinical problem (Kempe et al., 1962). In light of this study, and during the era when culture-of-poverty theory gained traction, policymakers and journalists increasingly defined child abuse (and later neglect) as a universal, classless social problem rooted in parental moral failure rather than economic deprivation (Nelson, 1986). Politically, a classless framing insulated child welfare from the controversies that engulfed welfare policy, allowing broad bipartisan support of child welfare programs that transcended disputes over welfare spending, race, and deservingness (Pelton, 1978; Nelson, 1986; Hacking, 1991; Gainsborough, 2010; Pelton, 2015). It also erased poverty as a central predictor of contact with the child welfare system, making invisible a well-established correlate of maltreatment allegations: poverty (Pelton, 1978, 2015). Thus, behavioral and moral scrutiny of poor families persisted under the language of universal child protection.

At the same time, the definitional boundary of child abuse shifted in policy and practice to include neglect, a far more elastic category and one more deeply entangled with poverty (Hacking, 1991). In fact, during the 1960s, U.S. state governments adopted mandatory reporting laws. These laws initially focused on physicians, but reporting requirements expanded in most states to include additional professionals, such as teachers, clergy, law enforcement, and in some states the general public. By the time our analysis period begins, neglect had become the dominant basis for child protective intervention, encompassing housing instability, insufficient childcare, inadequate supervision, and other conditions rooted in resource scarcity rather than parental neglect (Pelton, 1978; Nelson, 1986; Hacking, 1991; Pelton, 2015). This expansion disproportionately exposed poor families to surveillance and removal while preserving the rhetorical claim that child welfare was classless. As a result, poverty symptoms could be defined as endangerment. Failure to meet institutional expectations, missed appointments, lack of transportation, or refusal of mandated services could be construed as neglect rather than economic hardship (Reich, 2005; Fong, 2018, 2020). As a result, the number of children in foster care increased dramatically between the 1960s and 1990s (Roehrkasse, 2021).

### Policy shifts

These definitional and discursive shifts set the stage for the policy changes of the 1990s and beyond, which increased the likelihood that poverty-related hardship would be processed through child welfare systems rather than cash assistance. Prior to 1996, the primary federal program that provided cash assistance to poor families (mainly single mother families) was Aid to Families with Dependent Children (AFDC). In 1996, The Personal Responsibility and Work Opportunity Reconciliation Act replaced AFDC with Temporary Assistance for Needy Families (TANF), introducing strict time limits and lower cash benefits to reduce dependency and promote “self-sufficiency.” TANF rolls subsequently fell by roughly two-thirds between 1996 and 2019 (Pavetti et al., 2021). As a result, scholarly attention shifted from welfare receipt to life after welfare. Studies showed that TANF grant reductions resulted in higher rates of child maltreatment and child welfare interventions, a pattern linked to resource shortfalls and the stress they generate (Courtney et al., 2005; Pelton, 2015).

Just 1 year after TANF took effect, the Adoption and Safe Families Act of 1997 (ASFA) reoriented federal child welfare policy. The Act prioritized child safety over preserving biological families. In practice, poverty-related conditions, substandard housing, food insecurity, untreated medical needs, and missed appointments, were frequently defined as neglect, so the law accelerated adoptions while diminishing efforts to keep families intact (U.S. GAO, 2002). Noncompliance with service plans, missed drug tests, or perceived resistance to caseworker directives signified parental unfitness (Fong, 2018).

Prior to the passage of ASFA, federal law formally required agencies to make ‘reasonable efforts’ to prevent child removal and to reunify families, and policymakers frequently invoked ‘family preservation’ as an aspirational goal. In practice, however, foster care was consistently allocated far more resources than in-home services, and family preservation was often more rhetorical than real, particularly for poor families and families of color (U.S. GAO, 1993; Roberts, 2022). ASFA intensified a longstanding pattern: poverty-related conditions, substandard housing, and missed appointments were increasingly codified as neglect, and tighter timelines and adoption incentives shortened the window in which poor parents could demonstrate compliance. By equating poverty-related conditions with danger, it maintained a structural bias toward removal, even when families posed no physical threat, and brought child welfare policy into closer alignment with welfare reform’s behavioral and moral logic.

By the 2000s, research on the trauma of family separation, human trafficking risks, and racial bias prompted a partial policy correction. The Family First Prevention Services Act of 2018 encouraged states to fund programs to keep children with their families. Family First prioritizes prevention and is designed to help families address substance use, mental health issues, and parental stress in efforts to support families. Family First emphasized elements of family preservation, but it left the monitoring infrastructure in place. It also maintained the behavioral logic of prior reforms: families must demonstrate compliance with prescribed interventions to retain custody. Thus, the Act leaves intact the surveillance infrastructure that polices poor parents’ everyday lives (Fong, 2018, 2020; Randles and Woodward, 2025).

Together, welfare and child welfare reforms narrowed access to cash assistance while expanding the reach and discretion of child welfare systems and the touch points through which families could be introduced to and policed by the child welfare system (Roberts, 2002; Roberts, 2022; Falk, 2023). This created institutional conditions under which public and media discourse shifted from adult dependency to child maltreatment. In this policy environment, poverty-related hardship was increasingly governed through child welfare rather than cash welfare, expanding surveillance and discretionary intervention. While much of the current narrative around child welfare does not explicitly invoke cultural wording, the underlying perspective is maintained. Child maltreatment, often defined in terms of poverty symptoms, is treated as a pathology that must be transformed (Raz, 2020). The perceived lack of personal responsibility and parental failure is utilized to separate families.

### Revamped cultural theorizing

These institutional shifts unfolded alongside a new wave of cultural theorizing in relation to the poor. Some scholars frame child maltreatment as cultural adaptations to scarce resources that may be misinterpreted by individuals or organizations guided by mainstream cultural values. Here, what appears as ‘neglect’ to middle class gatekeepers often reflects pragmatic survival strategies—shared caregiving, episodic supervision, or discipline styles—shaped by structural constraints yet potentially misinterpreted through a mainstream lens (Keddell, 2023). For instance, child welfare systems attempt to involve children in case decisions, yet poor children may lack the cultural capital to communicate effectively, and their deference to authority can be misread as trauma (Keddell, 2023). Furthermore, children of color, socialized in more community-oriented norms, may also violate the individualist expectations of white middle class culture, leading practitioners to interpret communal caregiving as neglect (Keddell, 2023). Thus, recent cultural theorizing shifts attention from cultural deficits to classed and racialized interpretive frames.

Revamped cultural arguments also centralize structure. While original cultural arguments acknowledged yet underplayed the role of structure, new cultural arguments conceptualize culture(s) as an adaptation to structure. Historically, images of welfare recipients invoke frames aligned with individual deficiency while ignoring economic and institutional sources of hardship (Bullock et al., 2001). Relatively recent interviews with child welfare workers reveal that they see poverty as a mix of structural and individual forces (Hove et al., 2013; Carlson, 2016). Although some recent media coverage balances individual and structural explanations, it still tends to underplay poverty’s role, thereby perpetuating individualized accounts, accounts that are rooted in cultural interpretations (Bullock et al., 2001; Rose and Baumgartner, 2013; Madden et al., 2021).

### Research design

This study assesses media coverage of social welfare programs since the 1990s. To evaluate the framing of public welfare in newspaper articles, we utilized ProQuest’s U.S. Newsstream database to conduct a systematic search. The search included the following terms: “Unemployment benefits,” “Child welfare,” “Welfare reform,” “Public assistance programs,” and “Financial aid.” These terms were selected to capture the range of programs within the U.S. welfare state that support individuals and families in need. The search was restricted to articles published in news sources distributed in the United States and written in English. We selected three time periods for detailed analysis: 1994–1996, 2016–2018, and 2021–2023. The 1994–1996 period captures the years immediately prior to and during the passage of the Personal Responsibility and Work Opportunity Reconciliation Act (PRWORA), which created TANF. Utilizing this period as a point of comparison is insightful given its significance in welfare reform history and the extensive literature on welfare discourse during this era. The 2016–2018 period captures the years leading up to the passage of the Family First Prevention Services Act of 2018. Including the 2021–2023 period enables a comparison of public discourse before and after the COVID-19 pandemic, a time of dramatic shift in news coverage of social welfare programs (Mejia et al., 2022).

Importantly, the newspaper industry changed substantially during this period. Since 2004, approximately one-fourth of newspapers in the United States have vanished, and locally owned outlets have been replaced by large conglomerates (Abernathy, 2020). By 2018, 25 companies controlled two-thirds of all daily and one-fourth of all weekly newspapers, and the top 50 companies owned nearly half of all papers (Abernathy, 2018). This consolidation has produced widespread news deserts where local coverage is thin and most local news stories originate outside of the local market (Napoli et al., 2018). Many chains now operate a centralized content system that distributes standardized, nationally relevant stories to local outlets for publication (Uberti, 2017). Yet, while the overall number of newspapers declined during our study period, ProQuest expanded its indexing to include more local titles, many owned by national conglomerates that produce standardized content. Consequently, while our later datasets contain more local newspaper titles, the range of unique local reporting may be narrower. Our ProQuest searches returned 551 (1994–1996), 6,689 (2016–2018), and 4,974 (2021–2023) articles. After normalizing duplicate print-and-online editions (e.g., “Wall Street Journal” and “Wall Street Journal (Online)”), the search returned 50 unique outlets in 1994–1996, 269 in 2016–2018, and 208 in 2021–2023. The expansion of distinct outlets over time reflects both the proliferation of online regional editions and the inclusion of smaller metropolitan papers indexed in ProQuest. As a robustness check, we revisited the broad pattern using only outlets indexed in both the early and later periods and found that the overall pattern remained the same.

Our study is qualitative content analysis aimed at characterizing how coverage organizes meaning, namely, the dominant frames and their connections, rather than estimating population frequencies. We judge sample sufficiency by theme saturation and a clear, consistently applied sampling rationale (Moser and Korstjens, 2018). We set a target sample of 40 articles per period to enable close reading, with a pre-specified plan to expand the sample if new frames continued to emerge. In practice, new frames ceased to appear before the 40th article in each period, and the planned sample size proved sufficient to reach thematic saturation within each period. To avoid treating isolated mentions as meaningful patterns, we used a fixed within-sample benchmark for salience. Frames were interpreted as salient when they appeared in at least one-fifth of the sampled articles within a period. This threshold serves as a transparent decision rule within a qualitative content analysis. The articles were randomly selected to minimize researcher bias in article selection. Articles were randomly selected from the full set of ProQuest search results for each period (551 in 1994–1996, 6,689 in 2016–2018, and 4,974 in 2021–2023) using a random number generator to determine selection order.

In a few instances, articles were outside the scope of study and were replaced with the next article in the random sequence. This includes, for example, articles focused exclusively on disaster assistance, international events, or unrelated topics such as sports or finance. Because the sampling strategy was random, some geographic clustering occurred by chance. For example, six articles from the 2021–2023 period focus on Maine, reflecting intensified media attention following a series of high-profile child deaths. Thus, the sample captures major national and regional discourses.

The coding sheet was developed through an iterative process that combined deductive and inductive elements. Articles were coded in NVivo using an initial codebook adapted from Misra et al. (2003), which foregrounded dependency-oriented frames. To evaluate the fit of this framework and identify additional frames, the authors conducted multiple rounds of co-coding across the three periods. In each round, five articles from each period were jointly coded, and the codebook was refined to clarify definitions, collapse overlapping categories, and add emergent frames. This co-coding and revision process was repeated across approximately 15 articles in total (three to five articles per period per round) to finalize the frame list prior to coding the full set of 40 articles (see Appendix A). Once the frame list was considered exhaustive, intercoder reliability checks were conducted by having both authors independently code three articles per period. Discrepancies in coding were thoroughly discussed for each article, and full agreement on codes was reached. This process of co-coding three articles per period continued even after achieving 85% intercoder reliability to maintain a high standard of consistency. Intercoder reliability was calculated as percent agreement: the percent of codes on which both authors concurred prior to discussion. We report percent agreement rather than a chance-corrected statistic such as Cohen’s Kappa because codes were unevenly distributed across periods and units of text frequently received more than one code simultaneously, conditions under which chance-corrected statistics are less appropriate (Campbell et al., 2013). Approximately 75% of the articles in each period were discussed in detail to ensure coding agreement, while the remaining 25% were coded independently by the second author after 85% intercoder reliability was reached and sustained.

After coding, we identified the most prominent themes and examined their co-framing. We use NetworkX to visualize the thematic pairings (i.e., co-occurrences) surrounding each focal frame, such as dependency or child maltreatment. Each node represents a thematic code, and the figures display only those frames that co-occur in at least half of the articles coded for the focal frame. These visualizations are included as a transparency device to show that the interpretive patterns discussed in the text are grounded in recurring frame pairings within the sample rather than in isolated author judgments. They are descriptive and confirmatory within a qualitative content analysis and are not used for population inference.

After coding was complete, the authors noted the increasing salience of child maltreatment framing across periods and incorporated an abductive interpretive step, moving between the observed patterns and relevant theoretical literatures to develop an explanation for this shift (Timmermans and Tavory, 2012). This abductive component was interpretive rather than design-driven: the corpus and sampling frame were fixed in advance, and the analysis began from a dependency-centered framework adapted from Misra et al. (2003). Child maltreatment framing became analytically salient relative to this dependency-centered starting point only after patterns across the full coded sample were examined. It is important to note that throughout the analysis, we use the term child maltreatment to represent both child abuse and neglect as they were coded as a single theme.

## Results

Figure 1 displays the population of articles indexed under each search term in ProQuest between 1994 and 2024. Because articles could be associated with multiple search terms, the percentages exceed 100 percent. During the 1990s, a sizable proportion of articles focused on welfare reform—a trend that is unsurprising given the passage of the Temporary Assistance for Needy Families (TANF) program in 1996. However, attention to welfare declined over time, reaching a minimal level between 2006 and 2010, with a modest uptick in the 2010s and almost nonexistent coverage during the pandemic (2020–2021).

**Figure 1:**
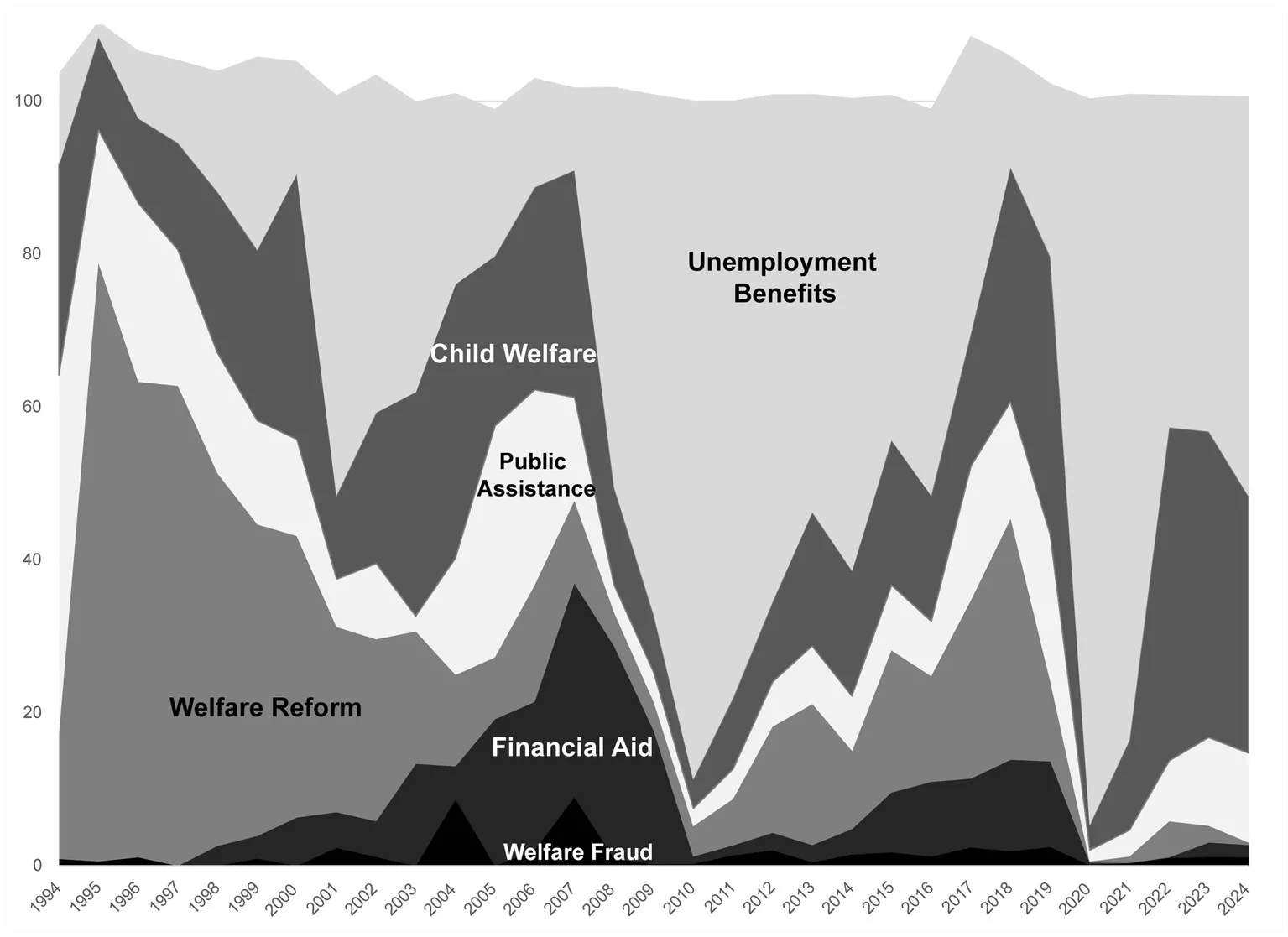
Distribution of articles in ProQuest.

Another major focus across the 1990s and early 2020s was unemployment benefits, which, unsurprisingly, fluctuated with economic conditions. Media discussions of unemployment benefits surged in the early 2000s as the U.S. economy faced shocks from the dot-com bubble collapse and the September 11 attacks. Unemployment benefits increasingly dominated coverage during the Great Recession, peaking in 2010, and again during the COVID-19 pandemic, peaking in 2020. Media attention to child welfare also grew steadily over time, with notable increases before the pandemic, coinciding with the passage of the Family First Prevention Services Act of 2018. Coverage nearly vanished during the pandemic, then rebounded and expanded in the post-pandemic period. Finally, media coverage of student financial aid was sparse before the mid-1990s, despite the 1965 Higher Education Act and the 1972 Pell Grant framing aid as an antipoverty tool. Attention grew in 1997, when the Taxpayer Relief Act shifted policy toward middle income families through new education tax credits. Coverage accelerated in the early 2000s and peaked in 2006 during debates that produced the College Cost Reduction and Access Act, which introduced income-driven repayment and Public Service Loan Forgiveness to protect low-income borrowers. Coverage fell during the Great Recession but climbed again after 2013 amid “student debt crisis” discussions, with a temporary decline during the pandemic, when most media coverage focused on unemployment.

Next, we turn to the programs discussed in the randomly selected articles. The sampled articles show the same broad shift visible in the larger ProQuest corpus, moving from a heavier focus on welfare in the 1990s to greater attention to child welfare and unemployment in the later periods (see Table 1). In the sampled articles, programmatic attention shifted sharply over time. The 1994–1996 period was dominated by welfare coverage, whereas the later periods turned more toward child welfare, unemployment, and student loan or school funding issues. In the most recent period, following the economic shock associated with COVID-19, about half of articles focused on unemployment or stimulus programs, and 38% on child welfare. Thus, the programmatic focus of our sampled articles reflects trends illustrated in the corpus of articles indexed in ProQuest, including shifting emphasis from public welfare to child welfare.

**Table 1:** Distribution of selected articles by primary program discussed.

Program	1994–1996	2016–2018	2021–2023
	*n* = 40	*n* = 40	*n* = 40
Child welfare	5%	23%	38%
Welfare	63%	5%	8%
Unemployment/stimulus	5%	23%	55%
Schools and loans	8%	30%	0%
Food stamps	8%	13%	3%
Other, including community programs	18%	18%	8%

Percentages sum to greater than 100 because some articles had a primary focus on more than one program.

### Cultural/individual frames

The frame analysis identified four frames associated with the culture of poverty thesis: dependency, out-of-wedlock births, drug use, and child maltreatment (see Table 2). In this study, percentages are used as a descriptive measure of recurrence to illustrate that our argument reflects patterns in the articles rather than an isolated mention. Unlike the population-level topic counts reported in Figure 1, the frame-level findings are drawn from the 120-article sample. They characterize which cultural frames were present and salient within this sample rather than estimate how frequently these frames occur across the full population of coverage. The coded frames show a clear reorganization of culture-of-poverty-related discourse across periods. Dependency is concentrated in the 1994–1996 sample, whereas child maltreatment becomes more prominent in the later periods. Dependency rhetoric was found in about one-third of articles (13 out of 40) in 1994–1996, zero articles in 2016–2018 and only 5% of articles (2 out of 40) post-pandemic. The framing of out-of-wedlock births followed a similar trajectory, discussed in one-fifth of articles (8 out of 40) in 1994–1996, zero pre-pandemic, and 5% (2 out of 40) post-pandemic. Drug use and child maltreatment were framed in 5% of articles (2 out of 40) in 1994–1996. The focus on drug use increased to 18% (7 out of 40) pre-pandemic and 10% post-pandemic (4 out of 40), and the focus on child maltreatment increased to 25% (10 out of 40) pre-pandemic and 30% (12 out of 40) post-pandemic. Given their broader salience in some time periods, dependency (1994–1996) and child maltreatment (2016–2018 and 2021–2023) are central in the discussion of culture of poverty related framing, while drug use and out-of-wedlock births are discussed as co-frames.

**Table 2:** Frequency of frames associated with culture/individual.

Frame	1994–1996	2016–2018	2021–2023
	*n* = 40	*n* = 40	*n* = 40
Dependency	33%	0%	5%
Out of wedlock births	20%	0%	5%
Drug use/abuse	5%	18%	10%
Child maltreatment	5%	25%	30%

### Dependency

Figures 2–7 present frame co-occurrence graphs. In each, the central node represents the frame anchoring that analysis (e.g., dependency, the economy, or child maltreatment), and surrounding nodes represent frames that co-occurred with it in at least half of the articles coded for that central frame. Frames with higher co-occurrence frequencies are positioned closer to the center node, with a thicker edge (i.e., connecting line) and larger circle (i.e., node). Edge labels give the exact co-occurrence proportion. Figure 2 visualizes the most recurrent thematic pairings surrounding dependency in the 1994–1996 sample.

**Figure 2:**
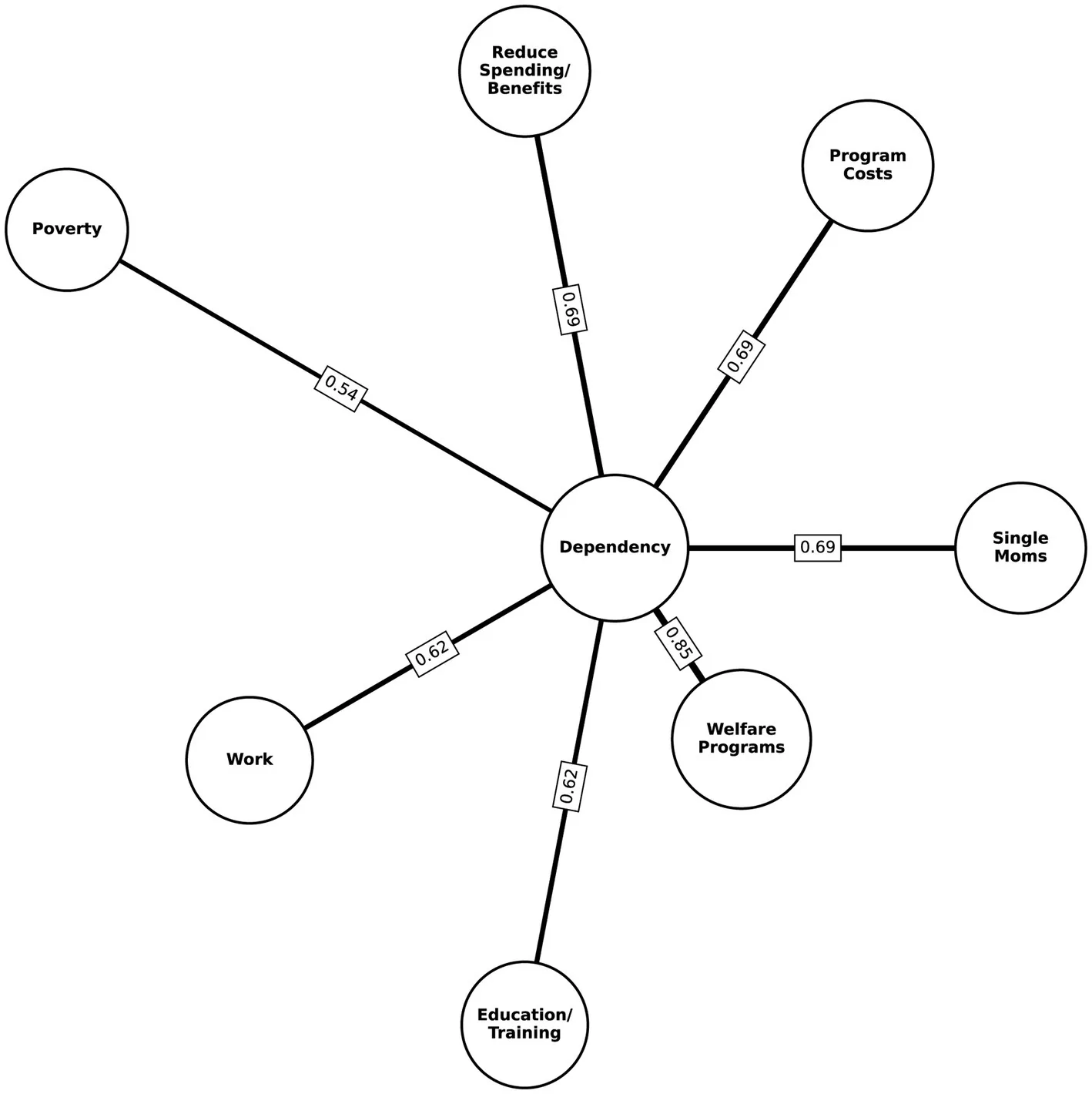
Dependency co-occurrence, 1994–1996.

**Figure 3:**
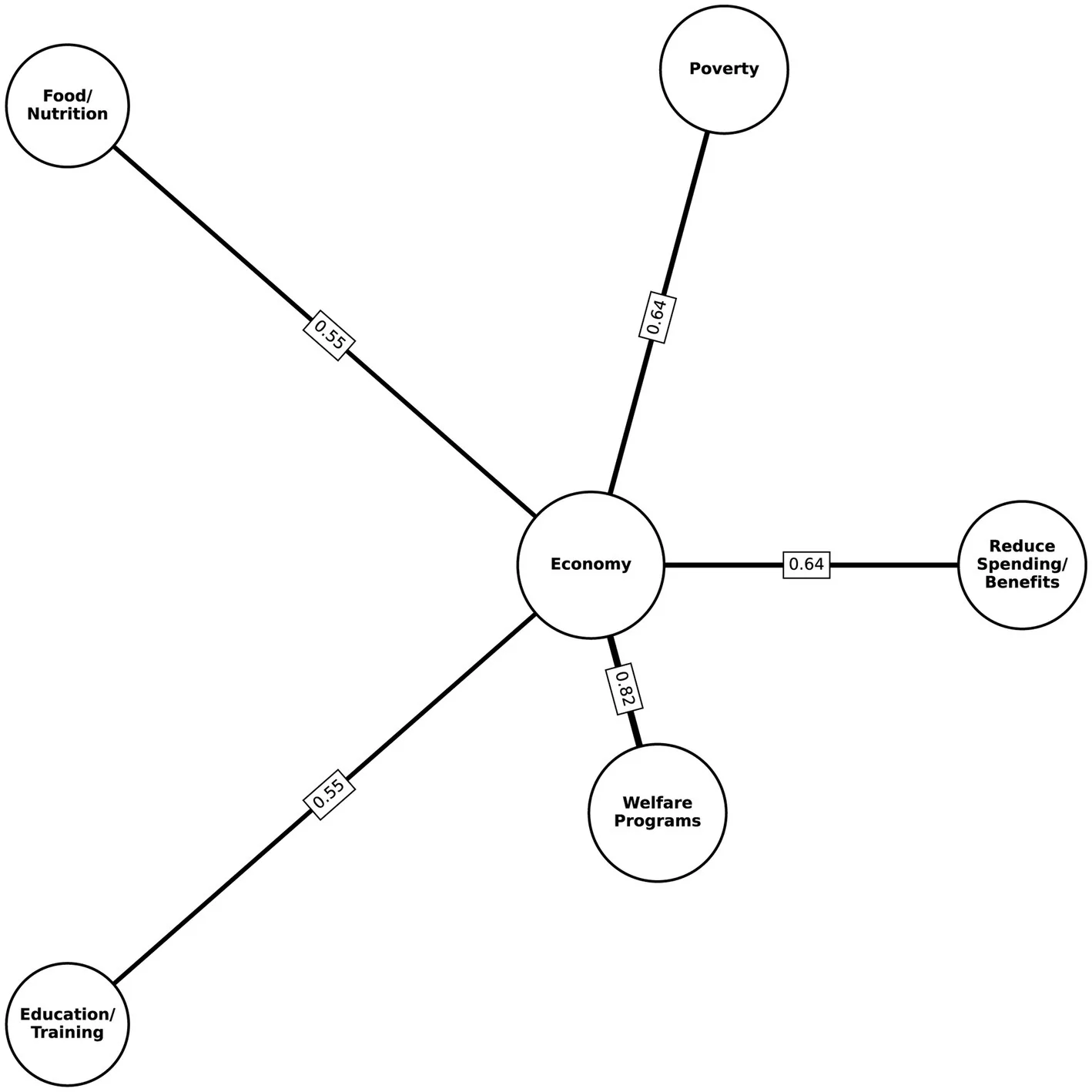
State of the economy co-occurrence, 1994–1996.

**Figure 4:**
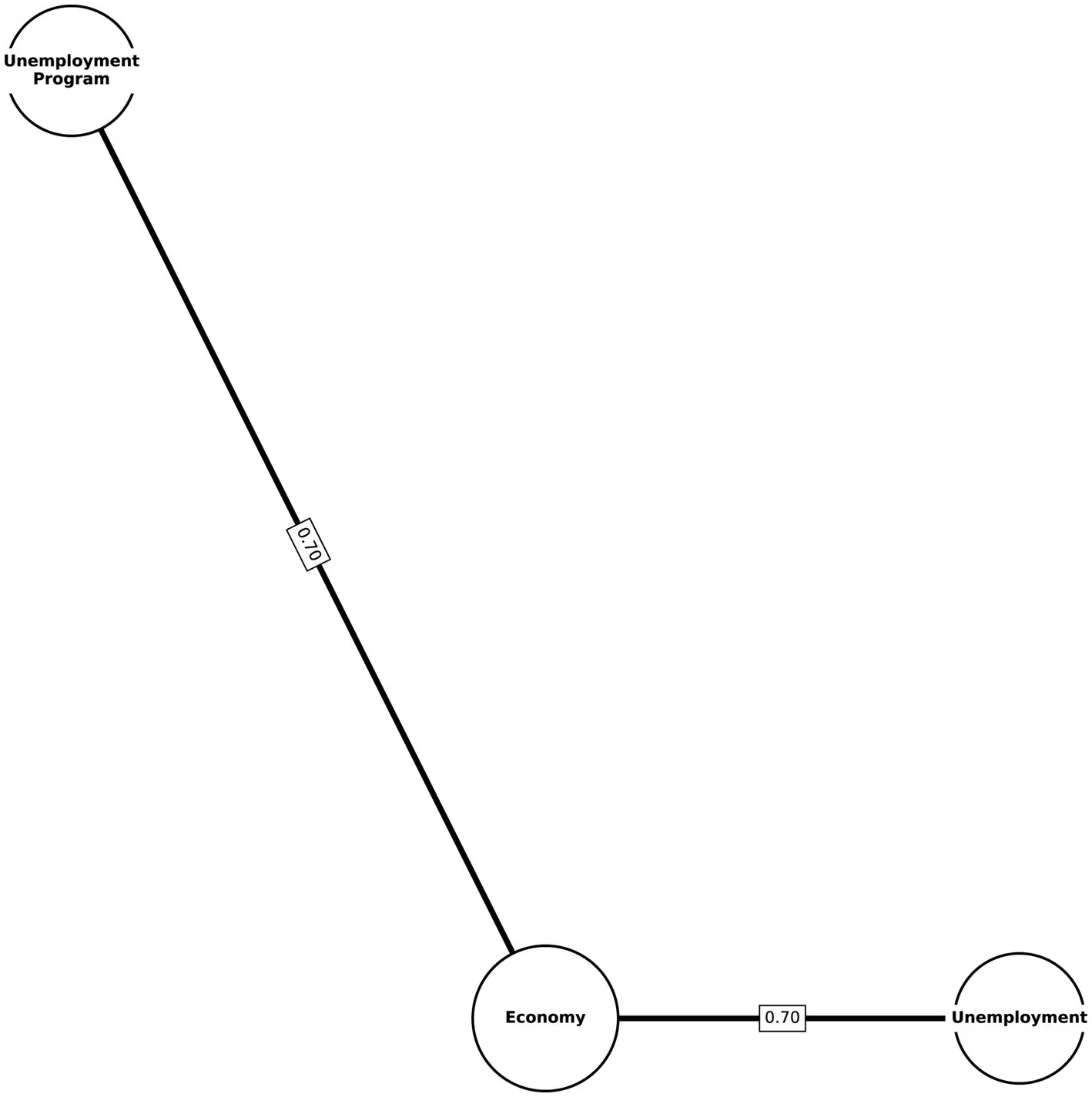
State of the economy co-occurrence, 2016–2018.

**Figure 5:**
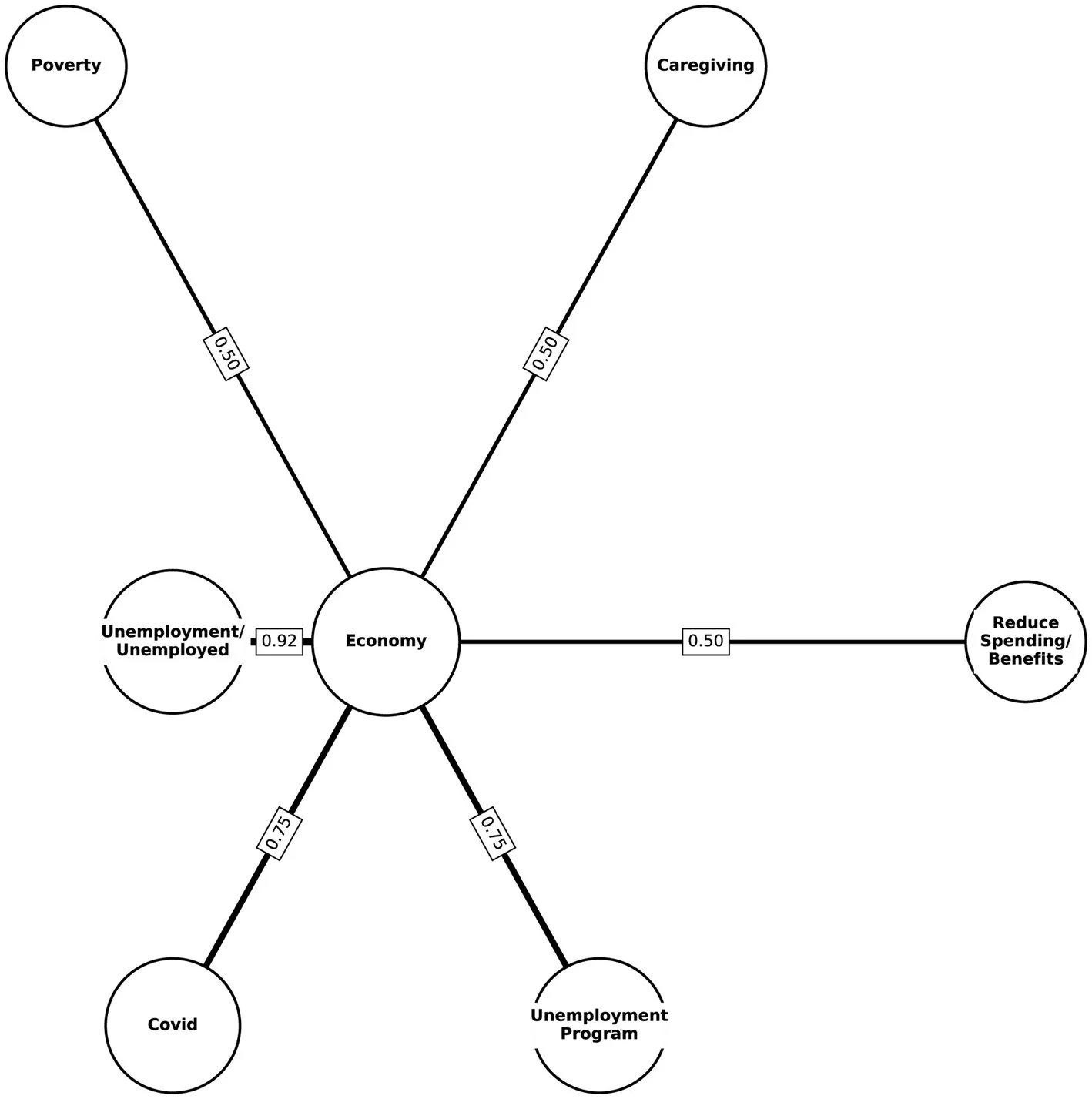
State of the economy co-occurrence, 2021–2023.

**Figure 6:**
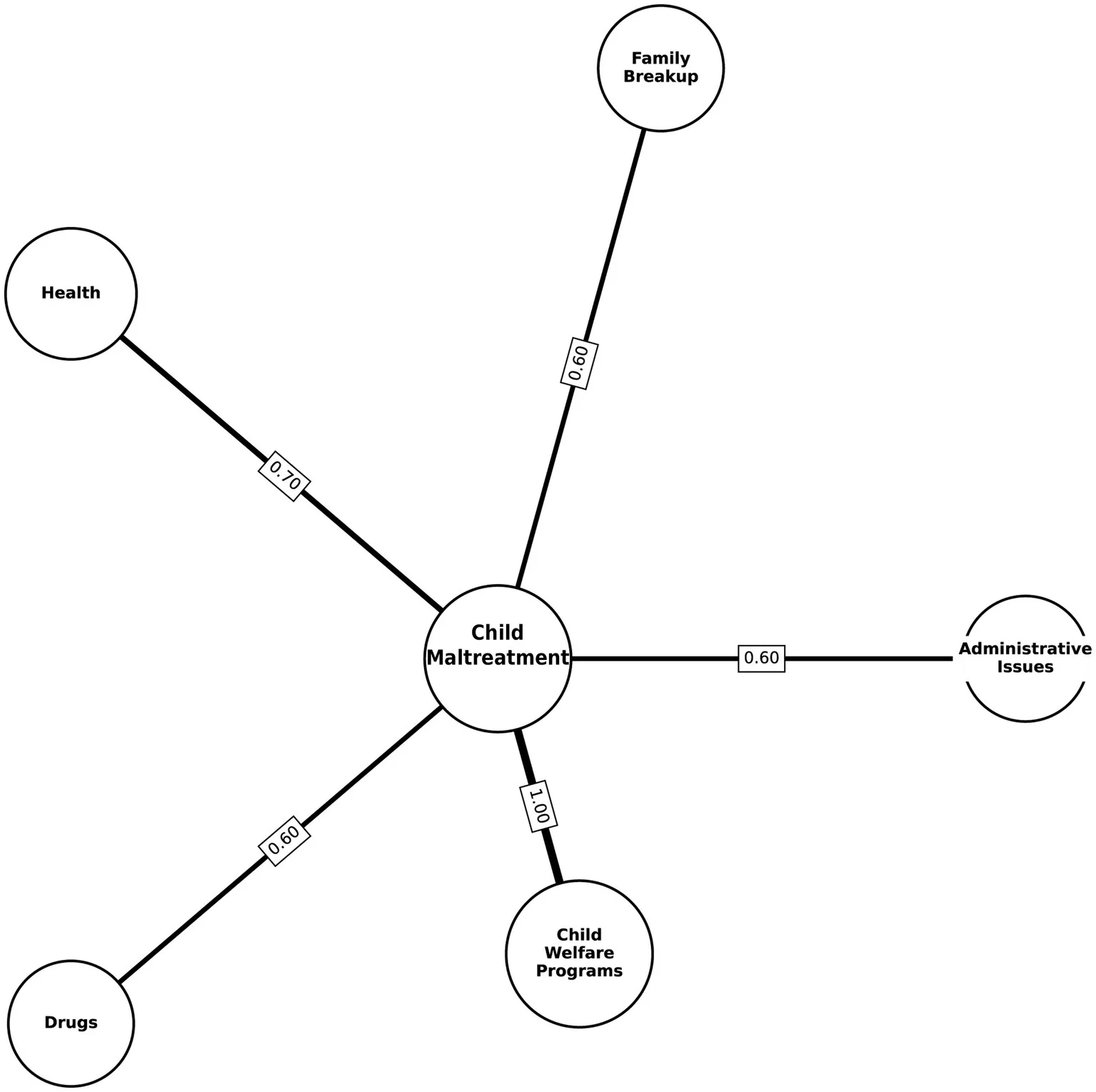
Child maltreatment co-occurrence, 2016–2018.

**Figure 7:**
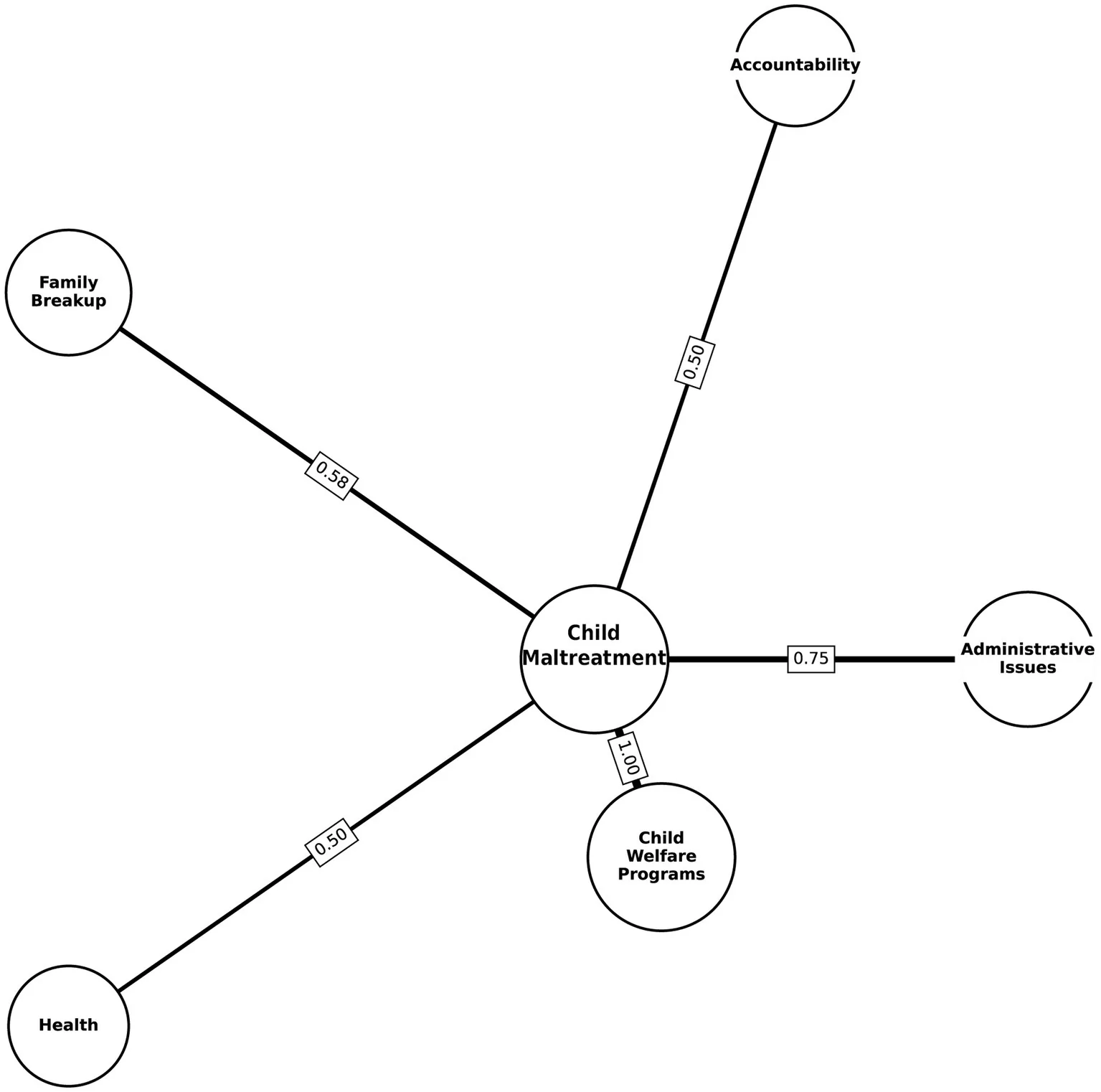
Child maltreatment co-occurrence, 2021–2023.

This visualization suggests that most articles that frame dependency are related to welfare programs, reinforcing the close association between dependency discourse and public assistance. In fact, as expected, none of the articles that discuss dependency also focus on unemployment programs. This aligns with previous scholarship that argues that modern conceptualizations of dependency rhetoric are aligned with public welfare and not broader programs to support the unemployed. We find that single moms are regularly mentioned in articles that also employ a dependency frame. Other frames that consistently emerged with dependency were reductions in benefits and services, cost and budgeting, and to a slightly lesser extent, work and education, and poverty and the poor.

The invocation of the dependency frame during the 1994–1996 period constructs the poor as deficient and lacking the behavior and values needed to achieve self-sufficiency. Articles suggested or quoted politicians who suggested that welfare reform proposals were designed to offer independence to the poor to make them whole. Articles directly stated that the poor lacked the values and morals of the broader society. A New York Times article (March 25, 1995) referred to political rhetoric that compared welfare recipients to animals who needed to be liberated from dependence: “Other Republicans, noting how animals can become dependent on people who feed them, said their bill would liberate people from dependence on Government Programs.” These statements illustrate the marginalizing and dehumanizing function of the dependency frame. Welfare recipients are portrayed not simply as in need, but as morally lacking or spiritually broken. This aligns with Entman’s (1993) conceptualization of framing as moral evaluation and Misra et al.’s (2003) analysis of moral coding embedded in dependency rhetoric. The comparison to animals and incomplete people reinforces stigma and casts recipients as less than human burdens on the state rather than citizens deserving of support.

The strong co-occurrence between dependency and single motherhood suggests how policy discourse locates the problem of poverty within the behavior of poor women, particularly young mothers. An article in The Atlanta Constitution (March 2, 1995) states, “In the dysfunctional American family, the teen mother may be the dependent, but by offering her money, however paltry the amount, the government becomes a codependent. Welfare may not promote her destructive behavior, but it certainly enables it.” This framing aligns with Fraser and Gordon’s (1994) argument that dependency functions as a moral keyword, used to distinguish between the deserving and undeserving poor. Articles suggest that welfare enables, rather than alleviates, socially problematic behavior. Notably, this framing appears not only among advocates of reform but also among its critics, who accept the premise that out of wedlock births signal moral decline—even when they also question whether the proposed policy changes will resolve it.

The 1994–1996 welfare reform bill emphasized time limits and benefit reductions as a strategy to end the so-called cycle of dependency. Accordingly, it is unsurprising that most articles invoking the dependency frame also addressed the costs of welfare programs and the distribution of benefits—two key points of contention among elected officials. For instance, State Assembly Speaker Sheldon Silver was quoted in a New York Times article (June 19, 1996), “I do not believe we should balance a budget entirely on the backs of the poor people of this state, as the Governor is.” Senator John Chafee, R-R.I., similarly criticized the House bill: “Instead of focusing on employability … out-of-wedlock births and … intergenerational welfare dependency, the focus (of the House bill) seems to be entirely on how to save money” (San Francisco Chronicle, April 3, 1995). These statements illustrate that critiques of welfare reform often remain tethered to the logic of the dependency frame. While some officials challenged the fairness or harshness of the proposed cuts, they did so by emphasizing the reforms’ failure to adequately confront the perceived root causes of dependency: intergenerational poverty and out-of-wedlock births. This again reflects what Fraser and Gordon (1994) identify as the moral function of dependency, used not simply to describe need but to assess worthiness. Even when framed as cost concerns, these debates rely on cultural assumptions about individual responsibility, family structure, and the appropriate role of the state.

Many of the articles that employed a dependency frame also discussed education and work, reflecting widespread agreement that employment and training are key to achieving independence. However, many of these articles simultaneously criticized policymakers for failing to adequately support these pathways out of poverty. A New York Times article (March 25, 1995) captured the prevailing moral framing by quoting Representative James M. Talent (R-MO): “the current system, while ‘trying to give people material wealth and lift them out of poverty, is luring them into a kind of spiritual poverty by destroying their families and their incentives to work.’” Similarly, Secretary of Health and Human Services Donna Shalala is quoted as defending welfare-to-work as it “reinforces the value of work and helps people move from dependence to independence” (Journal Record, March 14, 1995). Despite the emphasis on employment and self-reliance, many of the proposed reforms were criticized for failing to provide meaningful access to jobs, education, or training particularly during times of economic downturn. The same moral vocabulary that framed dependency as a problem of incentives and values, including “spiritual poverty,” structured the solution as individual behavioral change (work, independence). Media coverage reproduced the reform’s underlying premise even as it documented evidence against it.

Thus, some newspaper articles considered the state of the economy, a structural condition, when discussing dependency. Still, it is notable that only 23% of the articles that invoked the dependency frame also addressed the state of the economy (3 out of 13), a reminder that structural explanations remained largely peripheral in discourse that invokes the dependency frame. This aligns with prior research on media framing, which shows a tendency to individualize poverty and minimize the broader economic and institutional context (Gamson et al., 1992; Bullock et al., 2001; Misra et al., 2003).

Yet, a question remains. Did articles that did not employ the dependency frame invoke the economy frame? The 1994–1996 sample contained a distinct economy frame that did not fully overlap with dependency discourse. 28% of all articles sampled in 1994–1996 (11 out of 40) framed the state of the economy (27% of these also invoked the dependency frame). Figure 3 presents the co-occurrence graph for the economy during the 1994–1996 period. Most of the articles that discussed the state of the economy were also concerned with welfare, reductions in spending and benefits, poverty, education/job training and food/nutrition. The articles that frame the economy are most concerned about the role that the economy plays in reducing welfare rolls and the challenges that former welfare recipients (who met their time limits) will face when the economy weakens. Thus, during the 1994–1996 period, the media widely framed dependency, but it also widely framed the state of the economy and less than one-third of those had an overlapping frame. This suggests that there were competing frames that dominated discourse during the 1994–1996 period. This is highlighted in the following quote in The Sun (Baltimore, January 4, 1995): “Some link the problem to a breakdown of ‘values’ that has left the poor bereft of spiritual and moral compasses. Others see it as the result of a job market that no longer provides low-skilled workers with sufficient income.” This quote captures the era’s competing explanations: a moral account of poverty as personal or spiritual failing, set directly against a structural account rooted in the labor market.

By 2016, the discourse regarding the economy had completely shifted. Discussions of the economy in these articles no longer surrounded welfare. Instead, they were organized primarily around unemployment and unemployment programs, each of which appeared in most of the economy-coded articles (see Figure 4). These articles focused primarily on the rise or fall of unemployment claims and its relation to the state of the economy. There was little attempt to humanize or dehumanize the unemployed. The unemployed were discussed as a statistic designed to help readers understand whether the economy is strong, recovering, or weakening. The focus on unemployment during this period likely reflects the broader political context as 2016 was a presidential election year. Unemployment, economic insecurity, and candidates’ records on job growth were especially salient during the political campaigns.

By 2021, discourse surrounding the state of the economy changed in light of the economic shutdowns experienced during the pandemic. While a couple of articles continued to discuss the economy and its relationship with unemployment rates and unemployment benefits in a sterilized manner, the vast majority of articles that discussed the economy did so with a humanizing bent. Figure 5 illustrates that in the post-COVID period, the economy frame was closely tied to unemployment, COVID disruption, and household strain. These articles regularly discussed each of the following: poverty, childcare, and reducing benefits (primarily unemployment benefits). Articles asked why individuals left the labor market or what it would take to entice workers to return: citing the pandemic, fears of COVID, childcare demands, and unemployment benefits. These articles widely humanized the challenges that individuals faced, placing little blame on the unemployed or poor as they were simply seeking work-life balance and even satisfaction with their lives. This represented a shift in discourse. In 1994–1996, economic explanations for poverty existed alongside an individualizing moral frame that blamed dependency on the poor’s own values; by 2021 (the post-COVID period), discourse in the sampled articles shifted toward a humanizing treatment of economic hardship, largely free of the moralized blame that characterized the dependency era. Even as culture-of-poverty logic persisted and intensified in child maltreatment coverage over this same period, discourse about the economy moved in the opposite direction, away from individual blame. This divergence suggests the moralized, individualizing lens documented in this study was not applied uniformly across all poverty-adjacent topics, but became increasingly concentrated on parenting and child welfare specifically.

### Child maltreatment

We find that over time the media shifted cultural concerns from an emphasis on dependency to child maltreatment, while often retaining a similar moralized focus on parental deficiency. Returning to Table 2, the dependency frame disappeared completely in 2016–2018, with a slight rebound in 2021–2023. During 2021–2023, only two pieces touched the dependency frame. One used the classic formulation linking low income to reliance on benefits; the other debated whether expanded pandemic-era unemployment insurance reduced returns to work and whether its expiration would increase labor supply. At the same time, the focus on child maltreatment increased from 5% (2 out of 40) in 1994–1996 to 30% (12 out of 40) in 2021–2023. As noted earlier, these are within-sample contrasts, not population estimates. The shifting cultural framing of public discourse reflects a shift in policy emphasis from welfare in general to child welfare in particular. It also aligns with documented increases in investigations over the 2010–2019 period, albeit with a decline in investigations since 2020 (U.S. Department of Health and Human Services, Administration for Children and Families, Administration on Children, Youth and Families, Children's Bureau, 2016; U.S. Department of Health and Human Services, Administration for Children and Families, Administration on Children, Youth and Families, Children's Bureau, et al., 2022). This likely reflects the broader expansion of child welfare surveillance and mandated reporting that shapes when and how child maltreatment becomes newsworthy.

Returning to the distribution of themes in the full ProQuest corpus (Figure 1), the period in the early 1990s focused primarily on welfare reform, with little emphasis on child welfare, but this changed over time, as child welfare received sustained attention during the 2000s, with a sharp decline in 2008. By 2018, child welfare surpassed cash welfare in newspaper coverage, dropping dramatically during COVID, and rebounding post-COVID. Child welfare is specifically designed to protect children from harmful or abusive conditions.

Figures 6 and 7 present the co-occurrence matrix for the child maltreatment frame in 2016–2018 and 2021–2023, respectively. In both periods, all articles that framed their message in terms of child maltreatment also focused on child welfare programs. None of the articles that incorporate a child maltreatment frame are focused on unemployment programs, stimulus programs, or student loans. As previously mentioned, cultural arguments and perceptions related to child welfare and child maltreatment focus on the deservingness of families to remain intact, and they focus on maladaptive behaviors that are culturally rooted. We find that most articles that framed child maltreatment, both pre- and post-COVID, also included discussions of family dissolution. Further, scholars align this maltreatment with behaviors, such as drug use, that were defined as pathologies in early culture of poverty theories. The salience of drug use likely reflects the intensification of the opioid crisis in the mid-2010s, when rising overdose deaths and growing public recognition of opioid addiction as a national emergency pushed drug use and its effects on families to the center of news coverage. This context likely contributes to the tighter coupling of drug use and child maltreatment during this period. The co-framing of drug use was less salient post-pandemic. Other issues that are co-framed with child maltreatment are health (physical and mental), administrative issues, and accountability. These co-occurring issues extend the individualizing logic of the child maltreatment frame beyond parents themselves. Health co-framing often ties maladaptive parenting to physical or mental health conditions, framing harm as rooted in individual or family-level dysfunction rather than material hardship. Administrative issues and accountability, by contrast, direct scrutiny toward the child welfare system. This suggests that while child maltreatment coverage retained a strong individualizing focus on parents, it also shows a greater focus on institutional accountability than the dependency era did.

The framing of child maltreatment remains rooted in a narrative of individual deficiency, consistent with a culture of poverty perspective. For example, a private sector social work executive noted, “We really believe in the family, he said. ‘We think every family wakes up everyday and has no intentions of harming their kids, so we’re going to make sure they get a chance to get out of the system of care as fast as they possibly can, but this time with skills, relationships and connections to their own community. If they don’t have them, we’re going to build them for them.’” (El Paso Times, January 21, 2018). This statement reinforces the notion that parents are fundamentally deficient and require intervention from the state to learn appropriate parenting practices. This reproduces the individual blame frame rooted in culture of poverty rhetoric from the 1990s and extends it into child welfare (Pelton, 2015; Roberts, 2022).

A common co-frame during the 2016–2018 period is drug use and abuse, which was one of the original pathologies of poverty, as identified by Oscar Lewis. Drug abuse and neglect were cited as a common reason for removing children from their homes. For instance, one article emphasized that drug abuse surpassed neglect in 2016 as the leading cause of child removals in Minnesota. Another article, reporting on a child’s death attributed to maltreatment, emphasized how the child welfare system failed amid broader social issues like poverty, mental illness, and drug epidemics. Similarly, a 2021 article linked the accidental death of a child to systemic neglect despite years of agency involvement and referrals addressing substance abuse, mental health, and domestic violence.

While many of the articles recognized the central role that drug use plays in removing children from their homes, only one article stated explicitly the socialization component of drug use that underlies the culture of poverty perspective. “Children exposed to drug abuse often take on a lot of guilt and blame themselves, feel like they’ve got nothing to look forward to and believe their lives will always be bad, according to the National Alliance for Drug Endangered Children.” (Wausau Daily Herald, December 24, 2016). It is important to note that most articles did not explicitly state why drug use and the presence of drugs in the home are considered neglect. The articles simply stated, without critical reflection, that drug use and abuse were considered neglect.

One of the central co-frames during both time periods was family preservation versus separation. Articles examined reasons for removing children, efforts to prevent removals, and the importance of placing removed children with relatives or co-ethnic caregivers. It is clear across articles that the hierarchy of preference for children’s well-being was maintaining an intact family, followed by relative care, and finally foster parenting or adoption. Yet, the articles also portray an overburdened child welfare system. Some articles, particularly during the 2016–2018 period, actively sought to recruit foster parents.

The post-COVID period increasingly emphasized the trauma associated with removal decisions and considered whether these decisions were biased. Articles highlighted how removing children can cause emotional harm. However, the articles also recognized the potentially fatal consequences of keeping children in abusive homes, referencing multiple high profile child fatalities despite repeated warnings to child protection. These findings align with scholarly critiques emphasizing the delicate balance required in child welfare systems—protecting children from immediate harm without causing further trauma through overly aggressive interventions (Sankaran et al., 2018).

Given the widespread complications and challenges, administrative issues emerged as a common co-frame. During the 2016–2018 period, articles widely assessed administrative issues that undermined efforts to serve children. Journalists chronicled high caseloads, understaffing, low pay, caseworker training, and insufficient or unfollowed processes for child placement. As an example, a Tampa Bay Times article (April 25, 2017) about Florida’s child welfare system noted “The Florida Department of Children and Families plans to work with national experts to give investigators better training and to reduce their workload. There also will be more services for parents struggling with opioid and other addictions, more financial help for families who take in the children of relatives and more efforts to recruit foster parents.” The ability of social workers to adequately support families is a common frame that was regularly discussed in terms of training and caseload.

By the 2021–2023 period, these administrative issues were discussed alongside accountability. The articles that discussed accountability were mostly about child deaths in New Mexico and Maine. The articles expressed the importance of monitoring the child welfare system, ensuring transparency and holding the system accountable for the surges in child deaths.

Multiple articles during the 2021–2023 period discussed administrative issues and accountability in terms of lowering bias. One article that focused on a county in Pennsylvania emphasized the importance of accountability related to biased outcomes. “Research shows that in the United States, 1 in 3 children are investigated for abuse and neglect by the time they are 18 years old. For children of color, this number is 1 in 2. Nationally, black children are almost three times more likely to have some interaction with the child welfare system. Once referred to the child welfare system, black children are also more likely to be screened for investigation and placed in out-of-home care” (Morning Call, April 19, 2023). This is reiterated in a Los Angeles Times article (July 14, 2021), “‘For decades, Black and brown children have been substantially overrepresented in L.A. County’s child welfare system, and it’s past time for us to change that,’ said Supervisor Holly Mitchell, author of Tuesday’s motion. Seven percent of children in L.A. County are Black, while 24% of those removed from their homes from last April to this March were Black, according to the L.A. County Department of Children and Family Services. Twenty percent of children in the county are white, and 12% of those removed.” These articles reinforce Roberts’s (2022) claim that surveillance falls disproportionately on Black families. Other articles discussed issues associated with immigrant youth shelters, policies regarding LGBT parents and children, and ethnicity and foster placement.

Health emerged as a common co-frame with child maltreatment, in line with scholarship that links poverty to stress, mental health issues, and conditions that are perceived as neglect (Pelton, 2015). Many newspaper articles primarily highlighted the availability, or inadequacy, of health services, and several explicitly illustrated underlying issues. For instance, a Los Angeles Times article (May 10, 2018) criticized the push for speedy adoptions and described a scenario where an adoptive father, unable to handle a child’s undisclosed mental health issues, transferred custody to another family, where the child faced severe abuse. Poor health is a potential outcome of maltreatment and requires intervention.

### Additional frames

Beyond dependency and child maltreatment, the remaining articles were organized mainly around the economy, education and training, poverty and low income, and, in the most recent period, COVID-related hardship. When the economy frame is considered alongside dependency and child maltreatment, these recurring emphases account for just over half of the sampled articles across the three periods. Expanding the discussion to include education and training and poverty and low income captures most of the remaining articles, and adding COVID captures nearly all of the most recent period’s distinctive framing. In other words, once the two dominant cultural frames are set aside, the bulk of the remaining discussion turns to economic conditions, education, poverty, and pandemic-related disruption.

During the 1994–1996 period, education and training articles were heavily focused on welfare reform and impoverishment. By 2016–2018, the emphasis shifted: many of these articles centered on students, student loan programs, and program eligibility, often explaining how families could navigate loan applications and requirements. After the pandemic, the student loan focus largely disappeared. Instead, education was discussed alongside unemployment, poverty, caregiving, COVID, and stimulus payments. Many of these articles highlighted the strain on families educating children at home while also working or leaving the labor force.

The poverty and low-income frame was also present across all three periods, though its emphasis shifted over time. In 1994–1996, poverty discussions centered on welfare reform, program costs, and benefit cuts. By 2016–2018, the focus was more diffuse, with program eligibility emerging as the most consistent accompanying theme across welfare, energy assistance, and student aid. In 2021–2023, poverty discussions were more closely tied to COVID-related issues, including caregiving, unemployment, education and training, and debates over reducing stimulus payments. The pandemic thus recast welfare discourse around unemployment, associated programs, and the fiscal consequences of withdrawing relief.

## Discussion and conclusion

This research used a qualitative sample to examine newspaper articles in 1994–1996, 2016–2018, and 2021–2023, characterizing shifts in the cultural framing of welfare discourse rather than estimating their prevalence across the full population of U.S. news coverage. This study began with the widely studied 1994–1996 period because it anchors the analysis in prior findings and clarifies the pattern documented in the later periods. We found, in support of previous literature, that the dependency frame was salient in discussions of social welfare during the period of welfare reform in the 1990s. This corroborates an extensive literature that has found that dependency rhetoric remained a paradigmatic “individual-moral” frame in public discourse during this period (e.g., Rose and Baumgartner, 2013; Misra et al., 2003). This research also expands on prior studies by illustrating that the dependency frame becomes markedly less prominent by 2016, in part because the media shifted their focus from welfare in general to child welfare in particular.

We contend that stories of dangerous parenting display the same cultural repertoire that was previously attached to cash dependence, including moral suspicion, individual deficiency, and family level blame. Our findings suggest that the media did not discard the culture of poverty frame but rather reworked it. Long-standing narratives that pathologize poor parents have been re-routed through the language of child abuse and neglect as policy priorities and institutional practices have evolved. This evolution has taken place within a political landscape that historically disassociated child welfare from cash welfare in formal policy, while discreetly expanding the definition of child abuse to include neglect, with neglect defined as insufficient resources (e.g., poor housing conditions or insufficient childcare). Thus, dependency and child maltreatment rhetoric deploy the same logic while the specific ‘problem behavior’ evolves with policy priorities. This is consistent with social construction of target populations theory (Schneider and Ingram, 1993). The negative construction of poor parents is consistent over time, but the specific behavior invoked to justify it shifts. This pattern also speaks to framing theory more broadly (Entman, 1993). Rather than treating a frame as fixed to a single issue, our findings suggest a frame’s moral evaluation function can outlive its original topical anchor and reattach to a new one as policy priorities shift.

Across all three decades, news coverage portrays the state as failing poor families, first for not ending their “dependency,” later for not curbing parental behavior or removing children quickly enough. The media rarely acknowledges the structural engines of poverty that create both dependency and maltreatment. Only a handful of articles during the 1994–1996 period noted macroeconomic forces such as wage stagnation or deindustrialization, and even those mentions never became a sustained frame. Similarly, journalists in the 2000s recognized a widening drug epidemic, but they stopped short of probing its origins in chronic economic stress, social isolation, aggressive opioid marketing, lax prescribing oversight, and uneven state regulation (Kolodny et al., 2015; Dasgupta et al., 2018). Articles describe drug presence as automatic grounds for removal, rarely asking why parents self-medicate in the first place. In short, the media’s moral lens shifts from dependency to dangerous parents, but its focus remains fixed on individual shortcomings, leaving the structural roots of inequality and maltreatment virtually invisible and masking the culturally adaptive strategies that poor families employ to keep children safe under material constraints (Small et al., 2010; Keddell, 2023). By widely severing behavioral symptoms from their structural roots, newspaper articles tended to reinforce a parental pathology narrative without contextualization.

Several structural and journalistic factors likely contributed to the rise in child maltreatment coverage independent of the frame it takes: mandatory reporting requirements expanded the pool of documented cases, the opioid epidemic increased child welfare system involvement, high-profile child deaths drew sustained local coverage, and investigative journalism cycles periodically return to child welfare system failures. These factors can explain why child welfare coverage increased, but they do not explain why that coverage consistently moralizes parental behavior rather than emphasizing structural or systemic causes of maltreatment.

Even so, this analysis illustrates a shift in framing that aligns with shifts in the culture of poverty framework. Just as modern culture of poverty scholars argue that culturally rooted adaptations to poverty are often misinterpreted by mainstream society, media articles recognized cultural bias and misunderstandings as a source of concern and reason for reform. Thus, we argue that the culture of poverty perspective, and its focus on pathological behaviors of the poor that require state intervention, continues to influence media discussions of social welfare. However, public discourse invoking the perspective has shifted in alignment with theoretical advancements that centralize structure and recognize cultural bias.

Beyond dependency and child maltreatment, additional analysis highlights three widely discussed frames: education and training, poverty/low income, and pandemic-related unemployment. Their rise and fall mirrors contemporary policy debates: training and skills surfaced alongside 1990s welfare reform, student loan eligibility dominated the late 2010s, and COVID-era stories tied schooling to caregiving, job loss, and stimulus benefits. Poverty frames waxed in the mid-1990s, waned by 2016–2018, and rebounded once pandemic relief became contested. The economy itself remained a consistent frame, but its narrative focus shifted from program cost to labor force exits. These patterns suggest that when one dominant frame recedes, journalists do not converge on a single replacement; instead, they rotate among themes that echo the policy concerns of the moment. Yet, there is a consistent, though shifting, narrative that equates hardship with individual deficiencies, even in a shifting institutional landscape. Even so, discourse that invokes cultural frames has shifted in alignment with the academic literature, and there is a greater awareness of cultural heterogeneity and misinterpretation, but there is not a remarkable shift toward publicly discussing structural causes of the key issues facing poor children and their families.

Finally, we note that our latter two periods are relatively close together in time and straddle the COVID–19 pandemic. We selected 2016–2018 and 2021–2023 to ensure that our analysis speaks to the contemporary period and to assess whether the pandemic, as a major economic, social and institutional shock, was accompanied by a shift in welfare-related framing. Differences across these periods reflect both genuine shifts in media framing and shorter-term fluctuations in coverage. Empirically, we find that explicit attention to education declines in the post-COVID period, while child maltreatment remained a coherent and sustained frame even in the face of the pandemic.

As with all research, this study has limitations. The study population is limited by the breadth of newspapers indexed in ProQuest, and the database has expanded over time to include regional papers. The study is also limited by sample size. The sample is sufficient to find thematic saturation necessary for qualitative research, and a larger sample or perhaps even a machine-learning approach would allow for a more fine-grained picture to emerge. Another potential weakness of the study is that we initiated this research from a dependency framework, while also allowing other frames to emerge. We focused the ProQuest search on terms associated with social support as part of the broader welfare state (i.e., unemployment insurance, public welfare, child welfare, etc.). If we had started from a child maltreatment perspective, as opposed to a welfare perspective, then child maltreatment would have likely been salient in all periods. While this was not the focus of our study, future research could begin with child abuse, foster care, and child welfare to assess change over time specially tailored to these topics. In the meantime, we identify a clear shift within the sampled articles from dependency-centered welfare discourse to discourse organized around child welfare and child maltreatment.

Future research could also compare shifting discourse in the United States to discourse in other advanced welfare states to assess whether the frames found in U.S. media appear in other welfare regimes, where institutional designs and public problem definitions differ. Prior comparative research suggests media portrayals of welfare recipients and child welfare services vary systematically across national contexts and regime types (Larsen and Dejgaard, 2013; Schönfelder and Holmgaard, 2019; Lundberg and Syltevik, 2024), yet many of the existing studies do not consider the range of welfare state programs, or they focus on a specific component of reform such as poverty, immigration or the retirement age (Schönfelder and Holmgaard, 2019; van den Heijkant et al., 2024). Furthermore, nations have different media systems that influence journalistic standards and practices (Hallin and Mancini, 2004). Thus, cross-national analysis that focuses on a broad range of welfare state programs is a promising next step for distinguishing regime-specific dynamics.

Future research could also combine textual analysis with visual (photographic) analysis. This would permit a thorough investigation of racialized framing. Prior research found that race is often invoked in media images, as opposed to writing (Gilens, 1996; Clawson and Trice, 2000; van Doorn, 2015). Given norms against explicit racial language in mainstream coverage and in political discourse, racial meanings are frequently communicated implicitly through images rather than text (Entman and Rojecki, 2001; Mendelberg, 2001). Race was coded throughout the current study of text, and it became more visible in the later child welfare articles, where some coverage explicitly linked accountability and reform to racial bias. Future research could assess race framing visually.

The themes and patterns identified by this research offer conceptual insight into how welfare discourse has evolved in the recent time periods and point toward future research that could explore these framing patterns in larger samples. In our ProQuest indexed sample of newspaper articles, public discourse surrounding social welfare programs shifts from economic dependency to child maltreatment. We argue that this shift remains anchored in a cultural logic that links impoverishment to pathology. At the same time, more recent newspaper coverage begins to explicitly recognize structural sources of inequality. These findings do not claim to settle how widespread these frames are across the full universe of news coverage. Instead, we identified a shift that merits direct, systematic testing across larger and more diverse samples and that opens a clear agenda for future research.

## Data Availability

The raw data supporting the conclusions of this article will be made available by the authors, without undue reservation.
