# Supplementary material for: From dependency to child maltreatment? Changing frames in U.S. newspaper coverage of welfare policy
Source: Front Sociol. 2026 Sep 21;11:1860911. doi: 10.3389/fsoc.2026.1860911 (PMC13634873; doi:10.3389/fsoc.2026.1860911)
Supplement: Supplementary file 1 [file Supplementary_file_1.docx]

Appendix A. ProQuest Search Strings

(MAINSUBJECT.EXACT("Welfare reform") OR MAINSUBJECT.EXACT("Child welfare") OR MAINSUBJECT.EXACT("Unemployment benefits") OR MAINSUBJECT.EXACT("Welfare fraud") OR MAINSUBJECT.EXACT("Public assistance programs") OR MAINSUBJECT.EXACT("Financial aid")) AND (location.exact("United States US") AND stype.exact("Newspapers") AND la.exact("ENG") AND pd(19940101-19961231))

(MAINSUBJECT.EXACT("Welfare reform") OR MAINSUBJECT.EXACT("Child welfare") OR MAINSUBJECT.EXACT("Unemployment benefits") OR MAINSUBJECT.EXACT("Welfare fraud") OR MAINSUBJECT.EXACT("Public assistance programs") OR MAINSUBJECT.EXACT("Financial aid")) AND (location.exact("United States US") AND stype.exact("Newspapers") AND la.exact("ENG") AND pd(20160101-20181231))

(MAINSUBJECT.EXACT("Welfare reform") OR MAINSUBJECT.EXACT("Child welfare") OR MAINSUBJECT.EXACT("Unemployment benefits") OR MAINSUBJECT.EXACT("Welfare fraud") OR MAINSUBJECT.EXACT("Public assistance programs") OR MAINSUBJECT.EXACT("Financial aid")) AND (location.exact("United States US") AND stype.exact("Newspapers") AND la.exact("ENG") AND pd(20210101-20231231))

Appendix B. Codebook

***Category: Governmental Program or Service Discussed***

| **Code** | **Defining Terms (as coded)** | **Co-coding Rule** |
| --- | --- | --- |
| Social Security | Social Security | — |
| Unemployment | Unemployment, Jobless Claims | — |
| Cash Welfare | ADC, AFDC, TANF, Welfare Benefits, SSI | — |
| Health Coverage | Medicare, Medicaid | — |
| Food Assistance | Food Stamps, WIC, nutrition, lunch subsidies | — |
| Work Programs | Work Programs | — |
| Community Programs | Libraries, utilities, churches, etc. | — |
| Education Funding | University Funding, school funding, schools/education | Always co-coded with Issue: Education |
| Federal Grants | Federal Grants | — |
| Student Aid | Student Loans, FAFSA, Student Grants | — |
| Corporate Welfare | Corporate, Business Welfare, or Tax Breaks | — |
| Child Welfare Services | Child Welfare; Child Support; Child, Family Services; Foster, Orphanages | — |
| Stimulus Payments | Stimulus Payments | — |
| Other | (open code) | — |

***Category: Target Population Discussed***

| **Code** | **Defining Terms (as coded)** | **Co-coding Rule** |
| --- | --- | --- |
| Unemployed | Unemployed, out of labor force | Always co-coded with Issue: Unemployment |
| Teen/Unmarried Mothers | Teen or unmarried mothers | — |
| Disabled | Disabled, sick, unhealthy | — |
| Elderly | Elderly | — |
| Undeserving Stereotype | Lazy, cheats, criminals | — |
| Poor | Poor, low income, homeless | Always co-coded with Issue: Poverty |
| Generational Welfare | Generations on welfare; intergenerational | — |
| Minorities (general) | Minorities (not only African American) | — |
| African American | African American; Black | — |
| Immigrants | Immigrants, legal or illegal aliens | — |
| Childless Adults | Childless adults | — |
| Students | Students | — |
| Addicts | Addicts | — |
| Other | (open code) | — |

***Category: Issues***

| **Code** | **Defining Terms (as coded)** | **Co-coding Rule** |
| --- | --- | --- |
| Dependency | Dependency on the government, services, support | — |
| Fraud | Fraud, corruption, exploiting system (related to a service, program, spending or policy) | — |
| Theft/Crime | Theft, Crime (words must be used) | — |
| Child Abuse/Neglect | Child abuse, neglect | — |
| Out-of-Wedlock/Teen Births | Out of wedlock births, teen births | — |
| Drug Use | Drug use, abuse | — |
| Federal vs. State Control | Federal versus state oversight, control | — |
| Program Cost | Program cost (spending, budget, deficit, taxation, salaries, etc.) | — |
| Administrative Issues | Administrative issues, number of case workers, hard to navigate | — |
| Leadership Disputes | Leadership debates or disagreements or complications | — |
| Accountability | Accountability, transparency | — |
| Eligibility Requirements | Eligibility requirements | — |
| Universalism vs. Targeting | Universalism versus targeting, means-tested, entitlement | — |
| Benefit Reduction | Reduce, stop benefits to clients, services, supplies, time limits | — |
| Benefit Expansion | Increase, start benefits, services, supplies to clients | — |
| System Neglect | Doesn't do enough, doesn't get involved, blind eye (policy, program, service) | — |
| Family Breakup/Preservation | Family break-up or family preservation | — |
| State of Economy | State of economy (economic or job growth, decline, inflation, job market, interest rates) | — |
| Debt/Financial Crisis | Debt, debt crisis, financial crisis, financial difficulties | — |
| Caregiving | Childcare, eldercare, family care, caregiving | — |
| Poverty | Poverty, low income | Always co-coded with Population: Poor |
| Hunger | Hunger, food scarcity, food costs | — |
| Health | Health, healthcare, mental health | — |
| Race/Gender Equity | Race, sex or gender inequities / rights | — |
| Unemployment (issue) | Unemployment, nonemployment, OOL, labor market exit | — |
| Work Incentives | Worker incentives or disincentives, work requirements, work for benefits | — |
| Education | Education, work training, education accessibility | Always co-coded with Program: Education Funding |
| Corporate/Wealth Taxes | Corporate or business or wealthy taxes, welfare | — |
| Taxes (general) | Taxes (not corporate or wealth) | — |
| COVID/Pandemic | Covid, pandemic | — |
| Minimum Wage | Minimum wage | — |
| Immigration | Immigration | — |
| Other | (open code) | — |
